# Using Salivary Biomarkers for Stress Assessment in Offshore Saturation Diving: A Pilot Study

**DOI:** 10.3389/fphys.2021.791525

**Published:** 2021-11-30

**Authors:** Roxane Monnoyer, Jacky Lautridou, Sanjoy Deb, Astrid Hjelde, Ingrid Eftedal

**Affiliations:** ^1^Department of Circulation and Medical Imaging, Faculty of Medicine and Health Sciences, NTNU Norwegian University of Science and Technology, Trondheim, Norway; ^2^Centre for Nutraceuticals, School of Life Sciences, University of Westminster, London, United Kingdom; ^3^Faculty of Nursing and Health Sciences, Nord University, Bodø, Norway

**Keywords:** saliva, biomarkers, cytokine, inflammation, hyperbaric heliox saturation, physiological stress, hyperbaric hyperoxia

## Abstract

Health monitoring during offshore saturation diving is complicated due to restricted access to the divers, the desire to keep invasive procedures to a minimum, and limited opportunity for laboratory work onboard dive support vessels (DSV). In this pilot study, we examined whether measuring salivary biomarkrers in samples collected by the divers themselves might be a feasible approach to environmental stress assessment. Nine saturation divers were trained in the passive drool method for saliva collection and proceeded to collect samples at nine time points before, during, and after an offshore commercial saturation diving campaign. Samples collected within the hyperbaric living chambers were decompressed and stored frozen at −20°C onboard the DSV until they were shipped to land for analysis. Passive drool samples were collected without loss and assayed for a selection of salivary biomarkers: secretory immunoglobulin A (SIgA), C-reactive protein (CRP), tumor necrosis factor (TNF)-α, interleukins IL-6, IL-8, IL-1β, as well as cortisol and alpha-amylase. During the bottom phase of the hyperbaric saturation, SIgA, CRP, TNF-α, IL-8 and IL-1β increased significantly, whereas IL-6, cortisol and alpha-amylase were unchanged. All markers returned to pre-dive levels after the divers were decompressed back to surface pressure. We conclude that salivary biomarker analysis may be a feasible approach to stress assessment in offshore saturation diving. The results of our pilot test are consonant with an activation of the sympathetic nervous system related to systemic inflammation during hyperbaric and hyperoxic saturation.

## Introduction

Saturation diving is used for underwater work that requires direct human intervention. During saturation diving campaigns, the divers work and live in hyperbaric (pressurized) environments where they are constantly exposed to environmental stressors to which they must acclimatize (Brubakk et al., 2014). The human capacity to tolerate environmentally induced stress depends on a complex network of biological processes that serve to uphold homeostasis, some of which may be quantified *via* molecular biomarkers: biomolecules that correlate in abundance or state with the process in question (Godoy et al., 2018). Since biomarkers can also be used for risk prediction and to facilitate the diagnosis of environmentally induced disorders, they are frequently used in occupational health monitoring. But for biomarkers to be useful tools in offshore saturation diving, they must be safe and easy to obtain, robust to decompression, and not require advanced on-site laboratory processing.

Blood biomarkers are extensively used in clinical care. Blood biomarkers also reflect the oxidant-antioxidant balance involved in local and systemic pathologies, e.g., diabetes, cancer, inflammatory disorders, cardiovascular, and neurological conditions (Ho et al., 2013; Marseglia et al., 2014; Singh et al., 2019). With advancements in genomics and molecular biology, blood biomarkers of oxidative stress and inflammation have been studied in different types of diving, including saturation diving, to deepen the knowledge on the interactions between the immune system and the divers’ physiology (Eftedal et al., 2013; Sureda et al., 2014; Lautridou et al., 2016, 2017; Kiboub et al., 2018). However, phlebotomy is not an ideal procedure in saturation diving: it is difficult to perform under pressure, there is a small risk of infection, and a centrifuge is needed to prepare serum or plasma. Biomarkers are also present in other body fluids that are less challenging to obtain and prepare for analysis. One such fluid is saliva. Saliva is a readily available source of stress biomarkers, comprising of diverse chemical compounds, such as hormones (e.g., cortisol), enzymes (e.g., alpha-amylase), cytokines (e.g., TNF-α, IL-1β, IL-6, and IL-8), and immunoglobulins (e.g., secretory IgA) (Soo-Quee Koh and Choon-Huat Koh, 2007). Many studies have shown the interaction of the immune system and the central nervous system in stress responses (Hellhammer et al., 2009; Usui et al., 2011; Bozovic et al., 2013; Nater et al., 2013; Slavish et al., 2015; Dhama et al., 2019). This study aimed to examine whether salivary biomarker analysis might be a feasible approach for the assessment of environmental stress in offshore saturation diving. A panel of eight biomarkers was chosen to reflect stress responses in salivary glands and tissues, with emphasis on oxidative stress and inflammation which we hypothesized would increase due to the divers’ hyperbaric and hyperoxic work environment.

## Materials and Methods

### Ethics

The study material was collected during a commercial offshore saturation diving campaign in the Norwegian North Sea, in the fall of 2019. The protocol was approved by the Norwegian Regional Committee for Medical and Health Research Ethics (REK), approval number 2018/1184. The participants provided their informed consent prior to inclusion, and all procedures were conducted according to the Declaration of Helsinki principles for ethical human experimentation.

### Study Participants

Nine certified and experienced saturation divers, all male, were considered eligible and recruited as participants after passing a mandatory pre-saturation medical examination. The medical examination was performed in accordance with NORSOK U-100 Standards (Standards Norway, 2014) and ensured that the divers were currently in good health and fit to dive and committed to saturation onboard TechnipFMC’s DSV Deep Arctic. Anthropometric data for the participants are shown in Table 1.

**Table 1:** Study subject anthropometrics prior to saturation (n=9).

	Mean±SD
Age (years)	47±8.4
Height (cm)	180.4±7.4
Weight (kg)	89.0±10.2
BMI (kg/m^2^)	27.3±1.9

BMI=body mass index. The table is reprinted with permission from Deb et al. (2021).

### Saturation Diving

Heliox saturation diving was conducted according to NORSOK U-100 Standards (Standards Norway, 2014). The saturation system was compressed to an initial depth of 66 meters of seawater (msw), later increasing to 85 msw, for the divers to work at water depths of 74 – 93 msw. In addition to pressure control, the saturation chamber environment was continuously monitored and controlled by a life support crew to ensure that the divers’ living conditions adhered to NORSOK standards for gas mixture: partial pressure of O_2_ (ppO_2_)≤40kPa, pCO_2_≤1kPa, pCO<0.5Pa, relative humidity 40–60%, and temperature 22–33°C. The divers kept regular meals throughout the saturation, choosing their individual diets from daily selections from the vessel galley.

To facilitate continuous underwater activity, the divers were organized into four teams of three divers working overlapping 12-h shifts, with consecutive teams starting their shift every 6h. Each diver worked the same shift for the duration of his saturation. A pressurized diving bell was used to transport the dive team from the living chambers on the DSV to work on the ocean floor. During work excursions (bell-runs), the divers’ environment was monitored and controlled by diving supervisors *via* umbilicals from the DSV to maintain a breathing gas ppO2 of 60–80kPa, and hot water supply to the divers’ suits to preserve their body temperature. Daily bell-runs lasted for up to 8h, during which the divers spent a maximum of 6h in the water interspersed by breaks inside the bell for restitution and hydration. Their work consisted of installation of blind flange plugs, pipe support installation, seal replacements, bell mouth installation, and inspection work. The divers described the workload as relatively light compared to deeper and longer saturations, with most of their work performed standing on the ocean floor. At the end of their work assignment, the decompression back to ambient surface pressure lasted approximately 4days 4h, during which the ppO_2_ in the living chambers were kept at 50kPa until 13 msw, and gradually reduced to reach 21kPa at the end of the decompression.

### Saliva Collection

The participants were trained in the passive drool method and supplied with instructions for later reference before they proceeded to collect saliva into 2ml SalivaBio cryovials (Salimetrics, Carlsbad, CA); first during the pre-saturation medical examination (Day 0), then seven times during hyperbaric heliox saturation: three during the bottom phase (Days 1, 2, and 4) and four during decompression (Days 11, 12, 13, and 14), and finally post-saturation when the divers were back at surface pressure (Day 15). Samples in the pressure chambers were taken before bell-runs at the beginning of the divers’ work shifts, 1–2h after breakfast without brushing their teeth prior to the collection. Due to the continuous rotation with a new team starting their shift every 6h, each individual participant of the same diving team collected all his samples at the same time of day, whereas participants on successive teams did their sample collections at 6h intervals. The saliva samples were transferred out to the vessel hospital *via* a decompression lock, frozen at −20°C onboard the DSV and shipped frozen to NTNU at the end of the diving campaign. There the samples were stored at −80°C until they were shipped to Salimetrics’ SalivaLab (Carlsbad, CA) on dry ice for analysis.

### Salivary Biomarker Analysis

Samples were assayed in duplicate at the Salimetrics’ SalivaLab using the Salimetrics Salivary Assay Kits for C-Reactive Protein (CRP; Cat. No. 1-2,102), Secretory Immunoglobin A (SIgA; Cat. No. 1-1,602), Interleukins 1β (IL-1β), 6 (IL-6), 8 (IL-8), and Tumor necrosis factor alpha (TNF-α) (Salimetrics Cytokine Panel), Cortisol (Cat. No. 1-3,002), and Alpha-amylase (Cat. No. 1-1902), according to the manufacturer’s protocol. The assay data, with volumes, range, and sensitivity for each analyte, are shown in Supplementary Material.

### Statistics

**Figure 1:**
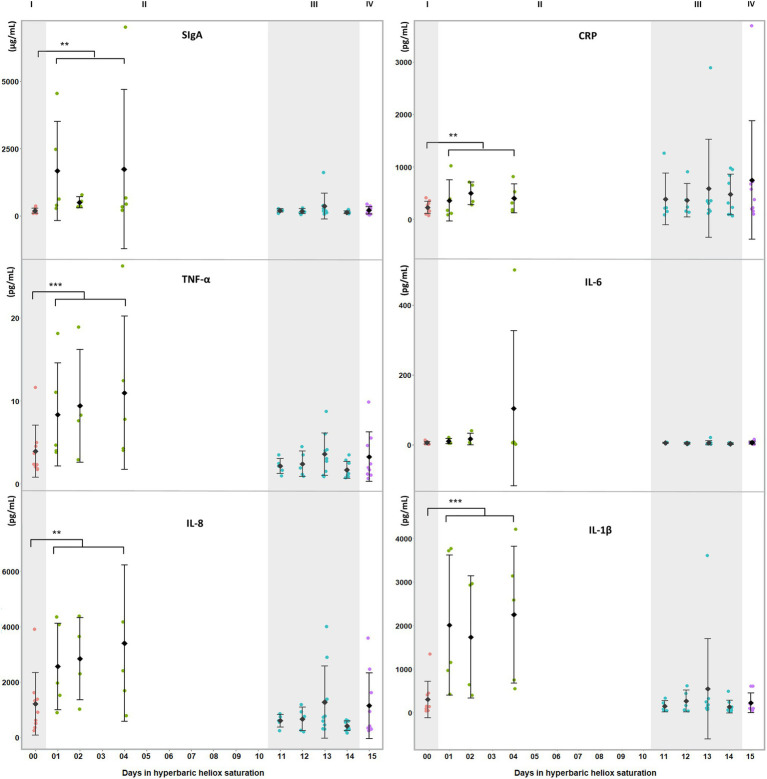
Salivary biomarkers of inflammatory and oxidative stress during a heliox saturation dive. For each time-point: mean and individual values are shown (*n*=9). Error bars are ±1 SD. ^**^, *p*≤0.01 and ^***^, *p*≤0.001. ANOVA was calculated on mean of days 1, 2, and 4 during the bottom phase and mean of day 11 to 14 during decompression. I (baseline: red dots); II (bottom phase: green dots); III (decompression: blue dots); and IV (post-saturation: purple dots). SIgA=secretory Immunoglobulin A; CRP=C-reactive protein; TNF-α=tumor necrosis factor alpha; IL-6=interleukin-6; IL-8=interleukin-8; and IL-1β=interleukin-1β.

**Figure 2:**
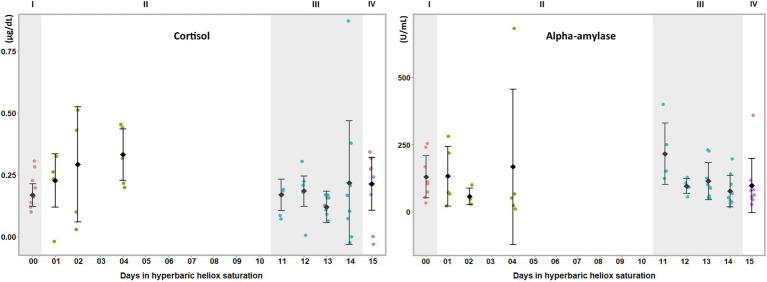
Salivary biomarkers of stress during a heliox saturation dive. Cortisol and alpha-amylase are reliable markers of HPA and ANS activity, respectively, in stress responses. For each time-point: mean and individual values are shown (*n*=9). Error bars are ±1 SD. ANOVA was calculated on mean of days 1, 2, and 4 during the bottom phase and mean of day 11 to 14 during decompression. I (baseline: red dots); II (bottom phase: green dots); III (decompression: blue dots); and IV (post-saturation: purple dots).

Prior to the statistical analysis, data from saliva samples obtained during saturation were split into two groups: one for the bottom phase and one for the decompression. Means were calculated at baseline (I in Figures 1, 2), bottom phase (II in Figures 1, 2), decompression (III in Figures 1, 2), and post-saturation (IV in Figures 1, 2). Statistical analysis was done in IBM SPSS Statistics software Version 26.0. After visual inspection for data distribution using Q-Q plots, the Shapiro–Wilk′s test for normality was calculated (*p*>0.05), either directly or after the data were transformed. A non-parametric analysis was applied if the data were still non-normal after transformation. For each biomarker, the differences within divers between the three time points were assessed by a one-way repeated measures ANOVA, with post-hoc Bonferroni adjustment for multiple comparisons. Differences were considered significant at *p*<0.05. Homogeneity of the data was assessed by the Mauchly test with *p*>0.05. If the assumption of sphericity was violated, a Greenhouse–Geisser correction was applied. Friedman’s test was applied for non-normal data, and pairwise comparisons were made with Bonferroni’s correction for multiple comparisons.

## Results

Salivary biomarkers were assayed before, during, and after offshore saturation diving. The saliva samples were collected by the divers themselves during a commercial diving campaign and transferred from the DSV to shore for analysis. The biomarkers were chosen to cover pro- and anti-inflammatory responses and oxidative stress: CRP, TNF-α, IL-6, IL-8, IL-1β, and SIgA as well as generalized stress responses: cortisol and alpha-amylase (Slavish et al., 2015; Dhama et al., 2019). The saliva collection proceeded without sample loss or technical issues, and the analysis yielded data that lay within the expected range for every biomarker analyzed.

During the bottom phase of the saturation dive, SIgA (*p*=0.005), CRP (*p*=0.005), TNF-α (*p*<0.0005), IL-8 (*p*=0.002), and IL-1β (*p*<0.0005) increased, whereas IL-6 was unchanged (Figure 1). There were no significant changes at any time in cortisol or alpha-amylase (Figure 2). All biomarkers returned to pre-dive baseline levels during or after the decompression.

## Discussion

In this study, we examined whether salivary biomarkers analysis would be a feasible approach for the assessment of environmentally induced stress in offshore saturation diving. In summary, we found passive drool collection performed by the divers themselves within the pressurized living chambers to be practical and to provide material that was suitable for analysis. Salivary levels of CRP, IL-1β, IL-8, SIgA, and TNF-α were elevated during the bottom phase of the hyperbaric saturation, whereas IL-6, cortisol, and alpha-amylase were unchanged. All changes observed during saturation were abolished at the end of the decompression.

The hyperbaric and hyperoxic environment in saturation diving induces pro-inflammatory responses which help the body adapt to the inherent oxidative stress in the maintenance of homeostasis. Prior work has shown that genes involved with the divers’ endogenous antioxidant defenses along with immune activity and inflammatory signaling pathways are upregulated at the time they complete their decompression from saturation (Kiboub et al., 2018). However, this knowledge was obtained from blood, and phlebotomy is not an ideal procedure to perform in hyperbaric chambers. Other body fluids, such as saliva, contain bioactive molecules that regulate inflammation and immune defenses (Cavalla et al., 2014) which have been recently used to evaluate the effects of saturation diving on measures of oxidative stress, including production of reactive oxygen species and total antioxidant capacity (Mrakic-Sposta et al., 2020). Saliva collection is non-invasive and requires no professional skills although passive drool collection requires prior training of the study subjects. Samples can be stored without pre-processing in a standard freezer (Wilde et al., 2013) or at room temperature with analyte stabilization (Bonne and Wong, 2012). Furthermore, saliva can be collected frequently, which makes it possible to semi-continuously monitor the effects of environmental exposure. Development of rapid tests may also facilitate real-time monitoring for some biomarkers, such as is already possible for salivary cortisol (Shirtcliff et al., 2015).

Saliva contains proteins and hormones from systemic sources *via* infiltration through the salivary tissues, and from local sources in the oral cavity *via* secretion by the acini and ducts of the salivary glands. The acini are responsible for the volume and flow of saliva, while the duct cells determine its composition (de Paula et al., 2017). The autonomic nervous system controls the function of both glands: Activation of the sympathetic nervous system triggers the production of protein-rich saliva at a low flow, whereas the parasympathetic nervous system causes the flow of liquids to increase, but results in saliva with lower protein content (Punj, 2018). SIgA is mainly secreted in the stroma of salivary glands by local plasma cells and stocked in the secretory epithelium (Brandtzaeg, 2013). Its secretion is governed by both parasympathetic and sympathetic afferences, with the latter having a stronger impact (Carpenter et al., 1998). IL-1β and TNF-α, most commonly known to be produced by activated macrophages, are also secreted by the salivary glands (Fernandez-Solari et al., 2010; Idris et al., 2015), whereas CRP and IL-8 have been hypothesized to enter the saliva *via* the gingival crevicular fluid (Out et al., 2012). The elevated levels of CRP and IL-8 in our study may be indicative of a systemic inflammation, especially since CRP does not originate from local tissues in the oral cavity (Megson et al., 2010). The activation of the sympathetic-adreno-medullar system in response to acute stress is measured by salivary alpha-amylase, whereas long-term or chronic stress is measured by salivary cortisol produced by the hypothalamic–pituitary–adrenal axis (Nater et al., 2013; Ali and Nater, 2020). Salivary alpha-amylase is also used as a marker of local inflammation in oral diseases (Gutierrez-Corrales et al., 2017).

Saturation diving can be mentally and physically stressful. In our study, inflammatory biomarkers IL-1β, TNF-α, SIgA, CRP, and IL-8 increased during the bottom phase of saturation diving. Conversely, there were no changes in IL-6, cortisol, and alpha-amylase. Although saturation diving is demanding, the physical workload in this campaign was reported to be light (Deb et al., 2021), and perceptions of stress and panic are unlikely to be prominent in experienced divers. IL-6 is known to increase in plasma in response to physical exercise, but this does not appear to be reflected in saliva (Cullen et al., 2015). Taken together, our results imply that the divers experienced an activation of the sympathetic nervous system secondary to systemic inflammation, rather than a generalized stress response. This is consistent with prior reports of oxidative stress and concomitant inflammatory responses in commercial saturation diving (Kiboub et al., 2018; Mrakic-Sposta et al., 2020).

### Limitations

The basal levels of salivary cortisol, SIgA, and alpha-amylase exhibit circadian variation (Stefaniak and Kaczmarek, 2013; Engeland et al., 2019), but due to the divers’ shift patterns and the limited number of participants, our study was not powered to account for this. However, circadian variation would cause inter-sample variation to increase, which is not expected to produce false positive results. Also, due to the low number of participants, we chose to merge samples taken during the bottom and decompressions phase, respectively, in the statistical analysis, thus forfeiting the option to monitor temporal development in biomarker expression.

### Conclusion

In conclusion, salivary biomarker analysis appears to be feasible as a non-invasive approach to environmental stress assessment in commercial saturation diving. The results of our pilot study are consonant with an activation of the sympathetic nervous system associated with systemic inflammation during the bottom phase, which was abolished by the time the divers completed the decompression.

## Data Availability Statement

The original contributions presented in the study are included in the article/Supplementary Material, further inquiries can be directed to the corresponding author.

## Ethics Statement

The studies involving human participants were reviewed and approved by Norwegian Regional Committee for Medical and Health Research Ethics (REK). The patients/participants provided their written informed consent to participate in this study.

## Author Contributions

RM, AH, SD, and IE designed the study. IE collected the material. RM and AH performed the statistical analysis. All authors collaborated on the interpretation of results and writing and approval of the final manuscript.

## Funding

This study is part of a Knowledge-Building Project for Industry, placed at NTNU, Norway and funded by the Norwegian Research Council and Equinor on behalf of PRSI Pool through the Large-scale Programme for Petroleum Research (PETROMAKS2), project no. 280425, *via* an integral part dedicated to research on Health, Safety, and Environment (HSE) in the petroleum sector.

## Conflict of Interest

Transport and boarding on the Deep Arctic for IE were sponsored by the TechnipFMC.

The remaining authors declare that the research was conducted in the absence of any commercial or financial relationships that could be construed as a potential conflict of interest.

## Publisher’s Note

All claims expressed in this article are solely those of the authors and do not necessarily represent those of their affiliated organizations, or those of the publisher, the editors and the reviewers. Any product that may be evaluated in this article, or claim that may be made by its manufacturer, is not guaranteed or endorsed by the publisher.
